# Evolution retraces its steps to advance

**DOI:** 10.7554/eLife.12386

**Published:** 2015-12-15

**Authors:** Daniel J Kliebenstein

**Affiliations:** Department of Plant Sciences, University of California,Davis, Davis, United Stateskliebenstein@ucdavis.edu

**Keywords:** evolutionary innovation, epistasis, experimental evolution, flux balance analysis, metabolic network, *E. coli*

## Abstract

Bacteria in a long-term evolution experiment evolved a new metabolic trait via two separate mutations with opposite effects.

**Related research article** Quandt EM, Gollihar J, Blount ZD, Ellington AD, Georgiou G, Barrick JE. 2015. Fine-tuning citrate synthase flux potentiates and refines metabolic innovation in the Lenski evolution experiment. *eLife* **4**:e09696. doi: 10.7554/eLife.09696

**Image** Evolving populations of *E. coli* have been tracked for almost 30 years (Image credit: Brian Baer and Neerja Hajela)

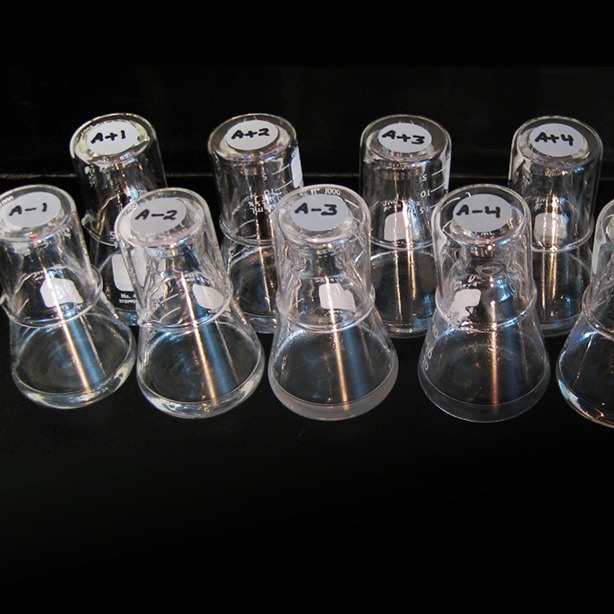

Selection can increase the fitness of a species in a stable environment by acting on random mutations. The same process can also create new traits if there is a change in the environment. Metabolism may evolve largely via the creation of new traits that either allow the organism to make use of new energy sources or provide new defense mechanisms in a complex environment (Blount et al. 2012; Prasad et al. 2012). However, we do not fully understand how new metabolic traits evolve or how they are integrated into existing metabolic networks.

Studying the creation of new traits is greatly complicated because evolution usually occurs over relatively long timescales. However, the Lenski long-term evolution experiment was designed to alleviate this problem and has been running at Michigan State University since 1988 (Fox and Lenski, 2015). Now, in eLife, Jeffrey Barrick and colleagues – including Erik Quandt as first author – make use of this resource to describe the molecular evolution of a new metabolic trait in *E. coli* (Quandt et al. 2015).

The long-term evolution experiment started with twelve identical populations of *E. coli.* These bacteria were forced to grow on culture medium that contained an excess of citrate, but very little glucose. Thus, for tens of thousands of generations of *E. coli*, the bacteria have been selected to evolve to use citrate as their main carbon source. This is something that *E. coli* would not normally do if they had access to oxygen. However, one of the populations did indeed evolve this exact ability (Blount et al. 2008; 2012). Sequencing the genome of this unique population throughout the long-term experiment identified the molecular changes that had generated this new trait. The new trait required two separate mutations within the gene that encodes an enzyme called citrate synthase (Quandt et al. 2015).

Barrick and colleagues – who are based at the University of Texas at Austin and Michigan State – now show that these two mutations have opposing effects (Quandt et al. 2015). The first mutation, called *gltA1*, abolished feedback inhibition in the enzyme and allowed the bacteria to use citrate, albeit weakly. This initial mutation was then followed by evolutionary shifts in genes that transcriptionally regulate primary metabolism (Leiby and Marx, 2014). Critically, this new transcriptional environment made the initial *gltA1* mutation detrimental to fitness which, in turn, led to the rapid selection of variants of the citrate synthase gene that made the enzyme less active. Thus, while two opposing mutations within a single gene were required, they had to occur in a specific order and this order caused the mutations to be positive in both instances.

These new results show that the apparently unwavering march of evolution towards a new trait hides a meandering process underneath. In particular, they show that mutations that were at one time beneficial can consequently become a drag on fitness, and that mutations within existing genes can allow the creation of a new metabolic trait. This is in contrast to the standard view that the creation of new genes, often by gene duplication, is essential to the evolution of new metabolic traits (Chae et al. 2014; Wisecaver et al. 2014).

The use of the long-term evolution experiment has illuminated the complex mechanisms that allow adaptation to a consistent selective pressure in a single direction. However, it is possible that fluctuating and unpredictable stresses in the environment are more important drivers of evolution in nature (Kerwin et al. 2015), so there is a need for long-term experiments that include such stresses. The work of Quandt et al. represents, I hope, only the beginning of our ability to empirically study evolution in action.
